# Chopped straw and coffee husks affect bedding chemical composition and the performance and foot pad condition of broiler chickens

**DOI:** 10.1038/s41598-023-33859-9

**Published:** 2023-04-23

**Authors:** Jakub Biesek, Mirosław Banaszak, Małgorzata Grabowicz, Sebastian Wlaźlak

**Affiliations:** Department of Animal Breeding and Nutrition, Faculty of Animal Breeding and Biology, PBS Bydgoszcz University of Science and Technology, Mazowiecka 28, 85-084 Bydgoszcz, Poland

**Keywords:** Environmental impact, Zoology

## Abstract

Bedding material is a crucial environmental factor for chickens. Coffee husks are waste from the industry that could be reused. The study aimed to analyze the chemical composition of various bedding types and assess their impact on the performance characteristics of broiler chickens and the incidence of footpad dermatitis (FPD). Ross 308 broilers were used in the study. Birds were divided into 3 groups (216 chickens, 72 per group). Group 1 was kept on chopped straw (S). Group 2 was kept on coffee husks (CHs), and the 3rd group (PB) was kept on pellet bedding made from S and CHs in a 1:1 ratio. The dry matter (DM), crude fiber (CF), nitrogen (N), phosphorus (P), potassium (K) content, and the pH of the bedding were analyzed. Production results were examined during 42 days of rearing. FPD was assessed on a point scale. The tissue composition of the carcasses and qualitative physicochemical characteristics of the meat (pH, color, water holding capacity, drip loss) and bone-breaking strength were analyzed. Straw had a higher CF content. In total, the highest N content in CHs was noticed. P content was lower in the S, and K was the highest in the CHs group, followed by S and PB. The pH of the bedding was lower in the CHs group, higher in PB, and highest in S bedding. The DM was decreasing within the days of rearing. A significant interaction was demonstrated between the type of bedding and rearing days on the bedding (manure) composition. On days 1–14, FCR deteriorated in the PB group compared to the S group. The presence of FPD was significantly lower in the PB group than in the others. In the PB group, chickens had a higher slaughter yield than in the S group and a lower weight and liver share than in the CHs group. The weight and proportion of abdominal fat were higher in the group kept on S than on CHs. It can be concluded that coffee husks as a component of pellets had a beneficial effect on reducing FPD in chickens and their slaughter yield and enriched bedding with nutrients, which with poultry manure, could be a good fertilizer for agricultural soils after rearing. It could be recommended to use pelleted bedding.

## Introduction

Poultry production is a dynamically developing animal farming sector that faces many challenges. First, the crucial is to obtain effective quantitative and qualitative results. In the production of broiler chickens, more and more attention is paid to their welfare^1^. Regarding welfare, footpad dermatitis (FPD) is a considerable problem in production management^2^. Contact dermatitis (pododermatitis) of the chicken foot pad is a disease favored by dynamic growth in a short time and inadequate quality of bedding^3^. According to Kuter et al.^4^, the increased incidence of FPD severity is also related to other factors, like heat stress. It adversely influenced the growth performance of chickens and bedding quality.

The bedding protects the chickens from high moisture or low floor temperature during rearing and should be able to absorb toxic compounds and excreta fractions^5,6^. The chemical composition of the bedding may affect its physical characteristics, e.g., absorption and moisture release. The fiber content is also essential. The fibers can regulate the specific absorption surface of the bedding. Bedding can also affect chickens' gut microbiota composition, growth, and health status^7^. After a few weeks of rearing, the bedding is covered with chicken manure, which promotes the development of microorganisms (bacteria, fungi, viruses, and parasites), as it is a good nutrient for them^8^. The high moisture content of the material can also increase the accumulation of ammonia (and nitrogen), which is related to the metabolism of microorganisms, and these would affect the welfare and productivity of birds^9^. It damages the foot pads due to constant contact with the wet bedding.

Subsequently, the epidermis of the foot pad undergoes keratolysis. It leads to necrotic lesions (superficial and deeper on the plantar surface of the foot pads and toes), ulceration, and, finally, FPD^10^. It reduces birds' comfort and affects their functioning, including movement and intake of feed and water^11^. Chicken feet with FPD symptoms result in financial losses, lower performance, and downgraded carcass quality^12,13^.

The study on the chemical composition of bedding collected from various housing systems found that bedding with a high level of nutrients (such as nitrogen, phosphorus, and potassium) is more suitable for use on agricultural soils as a fertilizer^14^.

A suitable bedding material should be chosen to avoid the problems mentioned above. Poland's frequently used cut cereal straw (rye or wheat) or pelleted material. A high moisture absorption characterizes the pelleted bedding. It is sterile (due to the production at high pressure and temperature)^15^. The beneficial effect of pelleted bedding was found^16^. The cited authors showed better properties of pellets than chopped straw and shredded paper. The FCR of birds kept on pelleted straw was improved, and a lower incidence of footpad lesions was noticed. Various bedding materials are used in different countries (for example, Brazil), including coffee husks (CHs). It relates to local opportunities and a highly developed coffee industry^17^. Coffee is one of the most widespread products. Its production is growing, and local coffee roasters are being established, also in Poland. The compounds in the husks characterize by their antimicrobial and anti-inflammatory activities and cytotoxic and protective properties for the nervous system. Flavonoids have beneficial effects on essential cell functions^18^.

CHs, costly waste to dispose of, threatened the environment, could be reused. CHs contain many nutrients, including elements that can be valuable fertilizers for plant crops after the chickens rearing (with manure)^19,20^. The rational management of waste from coffee production and other agricultural and food industries, including poultry manure, allows for environmental protection and sustainable development. The attempt to use different types of waste depends on their stability, production volume, and environmental impact. The literature indicates fewer possibilities for using CHs as a feed additive. There is also the possibility of using waste as bedding or in the energy industry (methane production)^21–25^.

Coffee processing is considered a growing industry. Moreover, detailed research is needed and impacts producers, the economy, people, and the environment^26^. The literature on using CHs as a bedding or pellet component is limited. Thus, the tested hypothesis is as follows: Different types of bedding materials, including chopped wheat straw and coffee husks, affect the chemical composition of the bedding during the rearing of broiler chickens, their performance characteristics, and the incidence of foot pad lesions.

The study aimed to analyze and compare the chemical composition of the bedding material, including chopped wheat straw, raw coffee husks, and a mixture of both materials (50:50) in the form of pellets, as well as to evaluate the performance characteristics, meat quality, and FPD in broiler chickens.

## Material and methods

The experiment followed the applicable regulations on protecting animals used for scientific or educational purposes. The study and methods were carried out after obtaining the opinion of the Departmental Animal Welfare Team and the permission of the Experimental Unit of the Bydgoszcz University of Science and Technology (No. 2/2022). The ethics committee of Bydgoszcz University of Science and Technology approved the experimental protocols presented in this study.

### Animals and experimental design

One-day-old Ross 308 broiler chickens were used in the study. Rearing lasted 42 days. The birds were divided into 3 equal groups. Each group was kept in 6 pens with 12 birds (216 chickens; 72 per group). The livestock density was max. 33 kg/1 m^2^. Each pen was 1.4 m^2^ (1 m^2^—usable surface; 0.4 m^2^ intended for devices). The randomization of the layout of the pens was preserved. The first group was on chopped wheat straw bedding (S), with approx. 5–7 cm of bedding depth. The second group was on raw coffee husks (CHs) with approx.—5 cm of bedding depth. The third group was on pelleted bedding (PB). The depth of the pellet bedding was approximately 1 cm. In each pen, 5 kg of pellets were used. The pellet was made of 50% chopped wheat straw and 50% CHs. The CHs came from a local coffee roaster (Kuyavian-Pomeranian Voivodeship, Poland). It was post-production waste. The pellet preparation was carried out under patent application pending procedure no. P.44383. The RTH-150 granulator (Pelleto, Poznań, Poland) was used.

The chickens were kept in a building where the temperature was 30 °C on day 1 and then lowered to 20 °C in the last week of rearing. For 4 weeks, the chickens were provided with an additional heat source with a temperature > 2 °C higher than in the building. The humidity was approx.—60%. The building was prepared (heating, bedding) 24 h before rearing. The lighting program was 18 h of light and 6 h of darkness. In the first and last 3 days of rearing, the lighting time was increased to 23 h. The chickens had access to fresh water and feed (ad libitum). A bell-shaped drinker and a feeder on the pen's walls were placed in each pen. The feed was purchased from a feed company. The feed was balanced according to broiler chicken nutrition standards^27^. Three feeding phases were considered: starter feed from day 1 to day 14, grower feed from day 15 to day 35, and finisher feed from day 36 to day 42 of rearing. Feeding was based on commercial feeds, so the standards followed the company’s recommendations.

### Chemical composition of bedding material

During rearing, bedding samples were collected into sterile string bags. Material from the pen's center, near its’ wall, and the feeder and drinker space was collected into one bag (2 bags per pen). The bedding material was collected on days 1 (fresh), 14, 35, and 42 (feed change and slaughter). Freshly collected samples were frozen until laboratory analysis commenced. The analyses were performed following the methods of the Polish Standards of the Polish Committee for Standardization (https://www.pkn.pl/). The dry matter content was determined using the PN-ISO 6496:2002 method (gravimetric method and pre-drying of the material). The mean value of the pre-drying factor was 1 (1st day, fresh bedding), 0.710 (14th day), 0.341 (35th day), and 0.440 (42nd day). The dry matter content was calculated based on this factor. Crude fiber content was determined by the PN-ISO 6965:2002 gravimetric method. The nitrogen content in the bedding material was analyzed using the Kjeldahl method (PN-EN ISO 6492:2005). Phosphorus was determined by the photometric method (Commission Regulation (EC) No. 152/2009 of January 27, 2009, Annex IIIP), and potassium by spectrophotometric atomic absorption (ASA, PN-EN ISO 6869:2002). The pH in the bedding material was measured using potentiometry. For each trait, 12 repetitions were performed within the group (6 pens with 2 repetitions).

### Growth performance

Chickens were weighed on the first day of rearing and then on the day of feed change (14th and 35th day). The last weighing of birds was performed before slaughter on the 42nd day. Based on body weight (BW), body weight gain $(BWG=final \; body \; weight- initial \; body \;weight (g)$) was calculated. The growth rate (%) was calculated based on the formula: $GR= \frac{(final \; body \; weight \left(g\right) - initial \; body \; weight (g))}{0.5 (initial \; body \;weight (g) + final \;body \;weight (g))}\times 100$.

Daily feed intake (FI) was monitored by weighing the feed and leftovers. Based on the data, the average daily weight gain $(ADBWG=\frac{BWG (g)}{days})$, the average daily feed intake $(ADFI= \frac{FI (g)}{days}$), as well as the feed conversion ratio per kg of body weight gain $(FCR= \frac{FI \left(kg\right)}{BWG \left(kg\right)})$ were calculated. During rearing, deaths were recorded, and the viability of birds in groups was calculated (%). Production efficiency indicators were calculated, including European Production Efficiency Factor ($EPEF=\frac{viability \left(\%\right)\times BW(kg)}{age(days)\times FCR(\frac{kg \; feed}{kg \; gain})}\times 100)$ and European Broiler Index $\left( {EBI = \frac{{viability\left( \% \right) \times ADG\left( {\frac{{\frac{{\text{g}}}{chick}}}{day}} \right)}}{{FCR\left( {\frac{{{\text{kg}}\;feed}}{{{\text{kg}}\;gain}}} \right) \times 10}}} \right)$. The methods and calculations were performed according to the descriptions of Biesek et al.^28^.

### Foot pads condition assessment

After rearing, the condition of the chickens' foot pads was assessed. The method was based on the Welfare Quality Project (based on photography from Welfare Quality, 2009, Butterworth, A. from the University of Bristol, North Somerset, UK), described by Rushen et al.^29^. The score scale ranged from 0 to 4. A score of 0 meant no visible skin changes, and a score of 1 and 2—meant minimal skin changes (hyperpigmentation, slight keratolysis of the foot pad, where on a scale of 1—spot on the foot pad. A score of 3 and 4 meant severe pododermatitis (erosions, ulcerations, keratolytic changes, discoloration, where on a scale of 4—changes overlap the foot pad and toes). One person was assessing to standardize the obtained results.

### Carcass yield and physicochemical features

On day 42, chickens were weighed, and 2 chickens from each pen with a body weight similar to the average within the pen were selected. Before slaughter, the birds had limited access to feed (8-h fasting). The slaughter was performed. The carcasses were scalded in water at 65 °C (10 s). Feathers were removed using an automatic plucker. The feet were cut off in the ankle joint, and the carcasses were gutted. The heart, liver, and gizzard were separated for further analysis. For cooling, the carcasses were placed in a refrigerator for 24 h at 4 °C.

Carcasses and offal were weighed (Radwag, Radom, Poland). Dissection was performed according to the method of Ziołecki and Doruchowski^30^. The neck (without skin), pectoral muscle (*m. pectoralis major* and *minor*), leg muscle (drumstick and thigh), skin with subcutaneous fat (with neck’ skin), abdominal fat, wings with skin, and carcass remains (trunk and leg bones) were cut. All of the elements were weighted. Slaughter yield was calculated $\left( {slaughter\;yield\left( \% \right) = \frac{{carcass\;weight\;\left( {\text{g}} \right)\;or\;elements\;\left( {\text{g}} \right)}}{{live\;body\;weight\left( {\text{g}} \right)\;or\;carcass\;weight\;\left( {\text{g}} \right)\;}} \times 100\% } \right)$.

Muscle tissue pH (in pectoral muscle) after 24 h was measured using a pH meter with a dagger electrode (Elmetron, Zabrze, Poland). Pectoral muscles (*pectoralis major* and *minor*) and leg muscles (thick and drumstick, boneless) were used to analyze physicochemical properties. The right leg bones (tibia and femur) were also collected for research. The color of the right pectoral and leg muscles (on the outside) was measured according to the CIE Lab method^31^. The values of L*-lightness, a*-redness, and b*-yellowness were determined. The right pectoral muscles were weighed and placed in string bags, and drip loss measurements were made^32^. The percentage of water loss was calculated. Left muscles were minced in a meat grinder (Hendi, Poznań, Poland) for water-holding capacity analysis (WHC) using the Grau and Hamm^33^ method.

The leg bones were weighed (right), and breaking strength was measured using an Instron 3345 (Instron, Buckinghamshire, UK) with Bluehill 3 software. The maximum force needed to break the tibia/femur bone (N) was measured. Instron Bend Fixture 10 mm Anvil and speed 250 mm/min were used. Relative bone-breaking strength (N/g) was calculated. Each feature was analyzed in 12 replicates per group.

### Statistical calculation

The obtained data were processed in the Statistica 13.3 program. (TIBCO, Statsoft, Krakow, Poland). The mean value in the group (S, CHs, PB) was calculated for each feature of the dependent variable. The standard error of the mean (SEM) was calculated. Data distribution (Kolmogorov–Smirnov test) and sample homogeneity (Levene’s test) were checked as a first step. Statistically significant differences were verified using a one-way analysis of variance. A post-hoc test (Tukey) was performed. Three groups were compared with each other. Six replicates were used for the production results, and 12 replicates for the bedding features and meat quality. Each bird’s foot pad scoring was calculated as a percentage per pen, so the 6 replicates were used per group. The significance of differences between the groups was assumed at a P-value < 0.05. For the bedding material's chemical composition and pH value, an interaction analysis (two-way analysis of variance procedure) was performed (bedding material × rearing day, 3 bedding materials × 4 days of rearing: 12 groups).

### Ethics

The experiment was carried out following the applicable regulations on the use of animals in science (directive no. 2010/63/EU, ARRIVE guidelines). The study and methods were carried out after obtaining the opinion of the Departmental Animal Welfare Team and the permission of the Experimental Unit of the Bydgoszcz University of Science and Technology (No. 2/2022).

## Results

### Chemical composition of bedding material

Table 1 presents data on the chemical composition and pH value of bedding made of chopped wheat straw, raw coffee husks, and pellets made of both materials in a 50:50 ratio, depending on the rearing day. Crude fiber (CF) content was significantly different depending on the type of bedding, in descending order: S > PB > CHs. Coffee husks had significantly higher nitrogen (N) content than the other groups. Group S showed the lowest N content, also compared to group PB. On the other hand, the phosphorus (P) content was significantly lower in group S than in groups CHs and PB. Potassium (K) content significantly differed between all groups, in order PB < S < CHs. Compared to the other groups, the highest pH value (close to alkaline) characterized the straw bedding (S). The CHs group had the lowest pH than the S and PB groups (P < 0.001).

**Table 1:** Chemical composition and pH value of bedding.

Group^1^	%^3^					pH
	DM	CF	N	P	K	
S	57.27	29.05^a^	3.11^b^	0.39^b^	1.32^b^	6.88^a^
CHs	56.94	22.10^c^	3.92^a^	0.47^a^	1.62^a^	6.58^c^
PB	58.98	25.37^b^	2.82^c^	0.44^a^	1.03^c^	6.71^b^
P-value (bedding)	0.132	< 0.001	< 0.001	< 0.001	< 0.001	< 0.001
Day 1	89.90^a^	33.17^a^	2.49^d^	0.13^d^	0.91^d^	7.00^a^
Day 14	67.10^b^	27.23^b^	2.77^c^	0.38^c^	1.19^c^	6.97^a^
Day 35	32.36^c^	21.96^c^	3.73^b^	0.56^b^	1.54^b^	5.95^b^
Day 42	41.55^d^	19.66^d^	4.14^a^	0.65^a^	1.66^a^	6.98^a^
P-value (days)	< 0.001	< 0.001	< 0.001	< 0.001	< 0.001	< 0.001
S × 1	90.86^a^	41.19^a^	2.98^d^	0.05^e^	0.76^e^	6.98^b^
CHs × 1	90.14^a^	29.25^c^	2.76^d^	0.08^e^	1.44^bc^	7.00^ab^
PB × 1	88.71^a^	29.07^c^	1.73^f^	0.26^d^	0.52^f^	7.01^ab^
S × 14	62.87^c^	33.69^b^	1.95^f^	0.31^d^	1.17^d^	6.92^b^
CHs × 14	60.32^c^	19.30^e^	3.99^b^	0.54^c^	1.69^a^	7.00^ab^
PB × 14	78.12^b^	28.71^c^	2.38^e^	0.30^d^	0.69^ef^	6.99^ab^
S × 35	36.48^ef^	20.67^e^	3.48^c^	0.57^bc^	1.72^a^	6.53^c^
CHs × 35	35.02^f^	20.52^e^	4.43^a^	0.57^bc^	1.59^ab^	5.40^e^
PB × 35	25.59^g^	24.69^d^	3.29^c^	0.54^c^	1.29^cd^	5.92^d^
S × 42	38.87^def^	20.65^e^	4.03^b^	0.62^abc^	1.61^ab^	7.11^a^
CHs × 42	42.27^def^	19.34^e^	4.51^a^	0.68^a^	1.77^a^	6.90^b^
PB × 42	43.51^de^	19.00^e^	3.87^b^	0.66^ab^	1.61^ab^	6.93^b^
SEM^2^	2.76	0.82	0.11	0.03	0.05	0.06
P-value (interaction)	< 0.001	< 0.001	< 0.001	< 0.001	< 0.001	< 0.001

^a,b^Different letters in the column indicate statistically significant differences between the groups, P-value < 0.05; ^1^, S, chopped wheat straw bedding; CHs, crude coffee husks bedding; PB, pelleted bedding with 50% of chopped wheat straw and 50% of coffee husks; ^2^, SEM, standard error of the mean; ^3^, DM, dry matter; CF, crude fiber; N, nitrogen; P, phosphorus; K, potassium.

When analyzing the bedding characteristics concerning the rearing days, a significantly higher dry matter (DM) content was found on day 1 and decreased within the next days (P < 0.001). Other features were highly significantly different (P < 0.001). CF content decreased successively with rearing days 1 > 14 > 35 > 42. In turn, the content of N, P, and K increased (day 1 < 14 < 35 < 42). The pH was significantly lower on the 35th day of rearing than on the other days.

Considering the influence of both factors, i.e., bedding and rearing days, significant differences (interaction) between the groups were found (P < 0.001). The highest DM content was found in all groups on the 1st day of rearing and the lowest in the PB group on the 35th day. However, the DM was the lowest considering interaction in all groups on the 35th and 42nd days (P < 0.001). The CF content in the group kept on chopped wheat straw (S) on day 1 was significantly the highest and the lowest in group CHs on day 14, in groups S and CHs on day 35, and in all groups (S, CHs, PB) on day 42. When analyzing the N content, its highest content was found in the CHs group on days 35 and 42 and the lowest in groups PB on day 1 and S on day 14. P content was the highest in the CHs group on day 42, quantitatively similar in groups S and PB on day 42, and the lowest in groups S and CHs on day 1. In turn, the highest K content was in the CHs group (day 42), S (day 35), CHs (day 14), and PB and S (day 42) sequentially. The significantly lowest P content was recorded in group PB on day 1. When comparing the significantly highest and lowest pH values, these differences were found between group S on day 42 (7.11) and CHs on day 35 (5.40).

### Growth performance of broiler chickens and footpad dermatitis assessment

When analyzing the production results of broiler chickens, a statistically significantly higher FCR in the first feeding phase (days 1–14) was found in group PB than in group S (P = 0.027). No significant differences were found in other features. The chickens showed a balanced growth performance (Table 2).

**Table 2:** Growth performance of broiler chickens.

Item^1^		Group^2^			SEM^3^	P-value
		S	CHs	PB		
Viability (%)		100.00	97.22	97.22	0.84	0.315
BW (g)	Day 1	45.63	45.81	45.56	0.13	0.737
	Day 14	371.90	362.94	331.26	7.65	0.065
	Day 35	1824.14	1813.07	1792.08	18.35	0.790
	Day 42	2463.56	2441.21	2426.55	28.32	0.879
The growth rate (%)	Days 1–14	156.19	155.06	151.18	0.99	0.089
	Days 15–35	132.28	133.30	137.66	1.00	0.054
	Days 36–42	29.87	29.51	29.95	0.64	0.962
	Total	192.72	192.61	192.61	0.08	0.837
BWG (g)	Days 1–14	326.28	317.14	285.70	7.66	0.067
	Days 15–35	1452.24	1450.13	1460.82	15.27	0.960
	Days 36–42	639.42	628.13	634.48	16.60	0.966
	Total	2417.93	2395.40	2381.00	28.29	0.879
ADBWG (g)	Days 1–14	23.31	22.65	20.41	0.55	0.067
	Days 15–35	69.15	69.05	69.56	0.73	0.960
	Days 36–42	91.35	89.73	90.64	2.37	0.966
	Total	57.57	57.03	56.69	0.67	0.879
FI (g)	Days 1–14	432.01	435.42	445.18	2.75	0.124
	Days 15–35	2213.26	2244.84	2247.74	15.17	0.617
	Days 36–42	1625.83	1660.20	1674.10	13.34	0.334
	Total	4271.11	4386.93	4407.05	36.38	0.272
ADFI (g)	Days 1–14	30.86	31.10	31.80	0.20	0.124
	Days 15–35	105.39	106.90	107.04	0.72	0.617
	Days 36–42	232.26	237.17	239.16	1.91	0.334
	Total	101.69	104.45	104.93	0.87	0.272
FCR (kg/kg)	Days 1–14	1.33^b^	1.38^ab^	1.58^a^	0.04	0.027
	Days 15–35	1.53	1.55	1.54	0.01	0.813
	Days 36–42	2.56	2.65	2.70	0.07	0.763
	Total	1.77	1.84	1.86	0.02	0.325
EPEF		332.27	309.61	304.85	8.05	0.352
EBI		326.12	303.81	299.14	7.95	0.355

^a,b^Different letters in the row indicate statistically significant differences between the groups, P-value < 0.05; letters are given from the highest ^(a)^ to the lowest value ^(…)^; ^1^, BW, body weight; BWG, body weight gain; ADBWG, average daily body weight gain; FI, feed intake; ADFI, average daily feed intake; FCR, feed conversion ratio; EPEF, European Production Efficiency Factor; EBI, European Broiler Index; ^2^, S, chopped wheat straw bedding; CHs, crude coffee husks bedding; PB, pelleted bedding with 50% of chopped wheat straw and 50% of coffee husks; ^3^, SEM, standard error of the mean.

Significant changes in the condition of the soles of broiler chickens' feet depending on the type of bedding used were shown (Table 3). In the group of chickens kept on PB, it was demonstrated that 52.40% did not show any skin lesions on the foot pads. On the other hand, significantly fewer birds in the CHs group had an 0 score (18.33%), and an even lower parameter was shown in the S group (5.68%). The differences were statistically significant (P = 0.005). In the group of chickens kept on straw bedding (S), the highest percentage (45.08% of the group) of birds with skin lesions on foot pads rated on a scale of 2 was shown, compared to group PB, in which the percentage of birds with a score of 2 was 5.32% (P = 0.005). In the remaining points, the beneficial effect of pellets was quantitatively demonstrated. There were no changes at the 3 and 4 points level in group PB compared to the other groups.

**Table 3:** Footpad dermatitis assessment of broiler chickens (% of the group).

FPD scale^1^	Group^2^			SEM^3^	P-value
	S	CHs	PB		
Points: 0	5.68^b^	18.33^b^	52.40^a^	6.68	0.005
Points: 1	28.28	33.61	40.66	4.46	0.553
Points: 2	45.08^a^	27.07^ab^	6.94^b^	5.32	0.005
Points: 3	18.18	19.47	0.00	5.20	0.243
Points: 4	2.78	1.52	0.00	0.78	0.365

^a,b^Different letters in the row indicate statistically significant differences between the groups, P-value < 0.05; letters are given from the highest ^(a)^ to the lowest value ^(…)^; ^1^, the method was based on the Welfare Quality Project (based on photography, Welfare Quality, 2009, Butterworth, A. from the University of Bristol, North Somerset, UK), described by Rushen et al.^29^; ^2^, S, chopped wheat straw bedding; CHs, crude coffee husks bedding; PB, pelleted bedding with 50% of chopped wheat straw and 50% of coffee husks; ^3^, SEM, standard error of the mean.

### Carcass yield, meat and bones features of broiler chickens

Table 4 presents the results regarding the tissue composition of the broiler chicken carcass and the slaughter yield. Significantly higher slaughter yield (carcass and carcass with offal) in group PB than in group S (P = 0.015; 0.021, respectively) was demonstrated. At the same time, pre-slaughter weight and carcasses were similar in all groups (P > 0.05). Higher liver weight and percentage in the carcass were found in the CHs group than in the PB group (P = 0.041). The abdominal fat weight and its share in the carcass were significantly higher in the group kept on chopped wheat straw (S) compared to the group kept on coffee husks (CHs) (P = 0.019; 0.010, respectively). No significant differences in other carcass characteristics were found (P > 0.05).

**Table 4:** Carcass yield of broiler chickens.

Item	Group^1^			SEM^2^	P-value
	S	CHs	PB		
Pre-slaughter body weight (g)*	2514.67	2489.82	2450.58	23.34	0.533
Carcass weight (g)	1821.00	1819.10	1818.50	17.19	0.998
Slaughter yield (%)	72.43^b^	73.05^ab^	74.23^a^	0.27	0.015
Carcass with offal weight (g)	1918.33	1917.44	1910.32	17.66	0.980
Slaughter yield (%) with offal	76.30^b^	77.01^ab^	77.98^a^	0.26	0.021
Heart (g)	13.51	13.07	12.78	0.25	0.507
Heart (%)	0.74	0.72	0.70	0.01	0.522
Liver (g)	54.55^ab^	58.31^a^	50.38^b^	1.30	0.041
Liver (%)	3.00^ab^	3.22^a^	2.77^b^	0.07	0.041
Gizzard (g)	29.27	26.96	28.66	0.76	0.455
Gizzard (%)	1.60	1.48	1.59	0.04	0.475
Neck (g)	60.22	61.86	59.35	1.38	0.769
Neck (%)	3.31	3.40	3.27	0.07	0.766
Pectoral muscle (g)	583.08	604.32	579.58	12.13	0.687
Pectoral muscle (%)	32.02	33.21	31.87	0.58	0.615
Leg muscle (g)	361.02	374.30	362.40	4.40	0.422
Leg muscle (%)	19.84	20.59	19.99	0.24	0.425
Total muscle (g)	944.10	978.62	941.98	13.29	0.474
Total muscle (%)	51.86	53.80	51.87	0.61	0.343
Skin with subcutaneous fat (g)	171.96	165.09	165.80	3.32	0.660
Skin with subcutaneous fat (%)	9.44	9.06	9.14	0.16	0.597
Abdominal fat (g)	23.63^a^	15.52^b^	20.93^ab^	1.22	0.019
Abdominal fat (%)	1.30^a^	0.84^b^	1.15^ab^	0.07	0.010
Fatness (g)	195.59	180.61	186.73	3.79	0.280
Fatness (%)	10.74	9.89	10.29	0.18	0.158
Wings with skin (g)	166.76	173.17	166.14	1.92	0.271
Wings with skin (%)	9.16	9.53	9.16	0.10	0.241
Carcass remains (g)	454.33	424.84	464.30	13.50	0.486
Carcass remains (%)	24.93	23.38	25.41	0.66	0.445

*The weight of chosen broilers for slaughter according to the methods; ^a,b^different letters in the row indicate statistically significant differences between the groups, P-value < 0.05; letters are given from the highest ^(a)^ to the lowest value ^(…)^; ^1^, S, chopped wheat straw bedding; CHs, crude coffee husks bedding; PB, pelleted bedding with 50% of chopped wheat straw and 50% of coffee husks; ^2^, SEM, standard error of the mean.

When analyzing the physicochemical characteristics of the pectoral and leg muscle (Table 5), no significant differences between the groups were found. The breaking strength of the tibia and femur bones was also similar in all groups (P > 0.05).

**Table 5:** Physicochemical features of broiler chicken muscles and bones.

Item^1^		Group^2^			SEM^3^	P-value
		S	CHs	PB		
Pectoral muscle	pH_24_	6.26	6.30	6.24	0.03	0.673
	L*-color	51.80	52.02	52.34	0.44	0.886
	a*-color	2.91	2.99	2.38	0.20	0.405
	b*-color	5.18	5.67	4.47	0.27	0.206
	Drip loss (%)	0.78	0.83	0.82	0.05	0.936
	Water holding capacity (%)	32.06	32.60	30.83	0.63	0.520
Leg muscle	L*-color	49.21	49.23	48.79	0.34	0.840
	a*-color	4.39	5.20	4.47	0.27	0.430
	b*-color	3.29	4.25	3.31	0.19	0.060
	Water holding capacity (%)	32.85	36.42	34.63	0.95	0.322
Weight of tibia bone (g)		13.31	11.89	12.98	0.28	0.101
Weight of femur bone (g)		18.46	18.25	17.21	0.46	0.499
Breaking strength of tibia bone (N)		231.98	224.08	202.71	13.66	0.670
Breaking strength of femur bone (N)		261.35	337.28	288.49	15.14	0.121
Relative breaking strength of tibia bone (N/g)		17.65	18.88	15.75	1.13	0.536
Relative breaking strength of femur bone (N/g)		14.50	18.46	17.09	0.86	0.161

No statistically significant differences between the groups were found, P-value > 0.05; ^1^, L*, lightness; a*, redness; b*, yellowness; ^2^, S, chopped wheat straw bedding; CHs, crude coffee husks bedding; PB, pelleted bedding with 50% of chopped wheat straw and 50% of coffee husks; ^2,^ SEM, standard error of the mean.

## Discussion

Our research showed the influence of the type of bedding material and the rearing period on the chemical composition of the bedding. The pelleted bedding characteristics were medium between chopped wheat straw and coffee husks because the pellet was made of both materials. A study by Potapov et al.^34^ dealt with reducing moisture from poultry waste, i.e., used bedding. The authors found that the humidity of the various bedding was different. The chemical composition of the bedding also depended on the type of chicken and the housing system. Omeira et al.^14^ showed that a higher content of N and P and a lower K content characterized the bedding in the intensive system compared to the free-range system. Manure, with high elements content, would be a beneficial solution for use in agricultural soils. Using raw CHs also showed a significantly higher content of N, P, and K at the end of the rearing period.

Souza et al.^35^ studied the quality of sawdust and coffee husk bedding with various chemical additives (superphosphate, gypsum, lime). Similarly to our research, coffee husks contained a higher percentage of N. Thus, the proposed bedding material is advantageous as a fertilizer after rearing. However, it should be mentioned that the N content changes as the bedding is stored^36^. In our research, the N content in the chopped wheat straw bedding decreased until the 14th day of rearing and then increased. It may indicate that the fresh bedding lost N, and its content increased due to the bedding mixing with manure and feed or feather residues^36^.

The alkaline pH of the tested bedding materials indicated their potential for fertilizer use^37^. The authors investigated the possibility of using biochar as an additive to composting poultry manure. The manure was mixed with biochar, CHs, and sawdust. The authors showed a much lower pH of raw CHs (4.81) than in our study (5.58). It may be related to their storage or the coffee type being processed. CHs have also been found to cause manure to degrade faster due to the presence of easily soluble carbohydrates. Our research also showed a lower fiber content in CHs, where soluble carbohydrates are included. Dias et al.^37^ found that CHs in bedding/manure may support the microbial activity of the material. It is essential during composting. Dalolio et al.^38^ reported in a literature review that the possibility of using coffee husk poultry bedding as an energy source was analyzed. The study on optimizing the mixing ratio of substrates for the wet alanine fermentation of selected organic waste streams for production biogas systems showed that the increase in the concentration of CHs in the chicken bedding had a positive effect on the nutrients of the fermenter. The C/N ratio (carbon to nitrogen) and the lignin concentration was improved. It may affect the digestibility of the substrate^39^. The significant changes in bedding DM within the days of rearing found in our research could be depended on the chicken manure excreted and the amount of water^40^.

In our study, the FCR in group PB was higher than in group S in the first period (days 1–14) of rearing. The FCR and FI parameters may be related to maintaining a constant body temperature^41^. Kheravii et al.^16^ compared the effect of various beddings (rice hulls, wood shavings, pelleted, straw, chopped straw, and shredded paper) on the growth performance of broilers. The authors found that only in the first rearing period (1–10 days) was the FCR affected, which corresponds with our results. It could be due to the bedding conditions and its consumption by the chickens in the early stage rearing period. After 2 weeks, the bedding was getting soiled and more compact. Straw formed a cohesive floor layer in the first days, influencing the birds’ comfort (floor temperature). Pellet structure may make it difficult for the birds to maintain the initial temperature. Feed consumption could have increased to keep the constant body temperature. It also may be related to the bedding depth. However, confirmation has yet to be found in the literature.

Bilgili et al.^12^ investigated the effect of different beddings (pine shavings, pine bark, chipped pine, mortar sand, ground hardwood pallets, chopped straw, ground door filler, and cotton-gin trash) on the presence of FPD. A relationship was found between the dry matter of the bedding and the condition of the chickens' feet. Wet litter is one of the major factors causing FPD in chickens^13^. The authors indicated the consequences regarding the formation of hock and breast burn and the reduction of bird welfare or even less effective production. Our research showed the lowest FPD incidence in the PB group, then on the CHs bedding, and the highest on the chopped wheat straw. Also, on day 42, there was a trend that PB had the highest quantitatively dry matter (43.27%), followed by CHs (42.27%) and S (38.87%).

The effects of peat moss and wheat-cut straw were also studied^42^. According to the authors' descriptions, straw was less favorable regarding FPD. Similar conclusions were reported by Farghly et al.^43^ comparing wheat straw to alternative materials (clover straw, cornstalk chips, sugarcane top chips, chopped palm spines, and corn ear husks). Lower FPD incidence in broilers kept on pellets was also reported by Kheravii et al.^16^. The other research examined standard-quality straw, low-quality straw, wood shavings, sawdust, and crop residues^44^. It was found that the bedding material significantly affected the footpad condition in broiler chickens. FPD may have been affected by the chemical reaction of ammonia combustion in the bedding, which is related to bedding moisture^6^. Raw CHs and pellets better absorbed and stabilized the excreted N. The advantage of using CHs may also affect the reduced presence of *Salmonella* spp. or *E. coli*^45^, compared with wood shavings. Thus, CHs have a potentially beneficial effect on the health status of birds.

Similarly, Zikic et al.^46^ studied the effect of chopped and unchopped wheat straw bedding on the presence of FPD. The authors showed that the lower FPD incidence was found in chickens kept on chopped bedding. It was affected by bedding quality and lower ammonia emissions. The relationship between the occurrence of FPD and bedding also concerned the thickness of the material and the cushioning properties. Appropriate bedding characteristics improve chicken production performance. In addition, the particle size of the bedding material was found to play an essential role in the development of FPD [^46,47^]. Thus, our research suggested that the lower incidence of FPD in chickens kept on CHs, especially pellets, was also due to their size compared to wheat-chopped straw.

In the group with raw CHs bedding, a higher liver weight and its share in the carcass were found. Similarly, the weight and proportion of abdominal fat in chickens on chopped wheat straw were higher than in the CHs group. Farghly et al.^43^ found that bedding material affects abdominal fat, drumsticks, and gizzard share. The association of higher gizzard weight with bedding intake and its type has been discussed. In our study, birds may have had a higher liver weight due to consuming CHs bedding. CHs have small amounts of tannins and caffeine. Webb and Fontenot^48^ showed a relationship between liver function in broiler chickens (accumulation of arsenic and copper) and birds’ bedding intake.

It also can be assumed that the differences in abdominal fat percentage in our study were related to the behavior of the birds and their activity (movement). The chickens may spend more time on the chopped straw. Higher abdominal fat in the body was deposited. Toghyani et al.^49^ studied the behavior of chickens depending on the type of bedding used (sand, wood shavings, paper, and rice husks). The authors found that activity was higher in chickens on rice husks. In production, abdominal fat deposition by chickens is problematic as fat can be lost during evisceration, affecting carcass yield^50^. Costa et al.^51^ indicated that a higher percentage of abdominal fat characterized birds kept on wood shavings (compared to rice straw), and the differences did not affect slaughter yield. In our research, despite the differences between the groups, no relationship was observed between the carcass yield and the abdominal fat percentage.

An economic study should have been conducted. However, the chopped wheat straw material was farm-sourced, and the CHs were obtained free of charge as waste. The only cost could relate to the electricity and labor during the production of pellets using our pellet machine. Kheravii et al.^16^ studied the potential of pelleted wheat straw as an alternative broiler bedding material. The authors concluded that pelleted bedding has potential benefits, but the high costs of pelleting could be considered uneconomic for production.

In conclusion, using raw CHs as bedding or a component to produce pellets in a 50:50 proportion with chopped wheat straw did not negatively affect the production results of broiler chickens. CHs bedding resulted in a lower share of abdominal fat in the carcass. The floor's temperature and the bedding's thickness should be considered. The beneficial effect of CHs, especially pellets, was shown to higher slaughter yield and reduce FPD, which is the essence of birds’ welfare and health status. Due to the rich nutritional composition (increased presence of N, P, and K) of CHs and poultry manure, there is potential to reuse waste from coffee production as a fertilizer for agricultural soils.

## Data Availability

The datasets analyzed during the current study are available from the corresponding author upon reasonable request. If there are some questions, the authors remain at your disposal.
